# Relationship between organizational climate and activities to prevent the risky use of alcohol, tobacco, and other drugs among professionals in primary health care

**DOI:** 10.1186/1940-0640-7-S1-A67

**Published:** 2012-10-09

**Authors:** Erica Cruvinel, Telmo Ronzani, Ronaldo Bastos

**Affiliations:** 1 Department of Social Psychology and Public Health, Federal University of Juiz de Fora, Juiz de Fora, Brazil; 2 Department of Psychology, Federal University of Juiz de Fora, Juiz de Fora, Brazil; 3 Department of Statistics, Federal University of Juiz de Fora, Juiz de Fora, Brazil

## 

Studies have shown that professionals working in organizations with a more positive organizational climate (OC) perform better at work. The aim of this study was to evaluate the association between preventive practices in relation to risky use of alcohol, tobacco, and other drugs and perceived OC among 149 professionals in Brazilian primary health-care (PHC) settings. The OC was measured by a scale involving the following factors: leadership, professional development, team spirit, relationship with the community, workplace safety, strategy, and reward. Prevention activities were measured by counting the number of drug and alcohol screenings conducted using the Alcohol, Smoking, and Substance involvement Screening Test (ASSIST) and the number of brief interventions (BIs) held within three months after theoretical training. We also used scales to examine self-efficacy and confidence in performing screening and BI and a structured questionnaire about drug-use prevention activities. To verify the proximity of the variables, we used multiple correspondence analysis and correlation analysis with 95% confidence intervals. The teams that had higher scores on OC also had the best performances in prevention activities for drug use. The OC factors most associated with performance of preventive activities were professional development and relationship with the community. The dimensions of leadership and rewards also showed significant positive associations. Findings suggest that a more positive OC can facilitate drug-use prevention activities in PHC settings.

